# The potential for biosimilars to address unmet clinical need — Is the developed world missing out?

**DOI:** 10.1515/rir-2024-0027

**Published:** 2025-01-09

**Authors:** Whei Chuern Yeoh, Deva Situnayake, Robert J Moots

**Affiliations:** Department of Rheumatology, Liverpool University Hospital NHS Foundation Trust, Liverpool, United Kingdom; Department of Rheumatology, Sandwell and West Birmingham Hospitals, Birmingham United Kingdom; Faculty of Health, Care and Medicine, Edge Hill University, Ormskirk, United Kingdom

Biologics, a diverse group of medicines, typically protein-based and manufactured through recombinant DNA technology, have been used in one form or another since the early 1980 s and play important therapeutic roles in many medical conditions worldwide, from rheumatic diseases to malignancy.^[1]^ Since their introduction in rheumatic diseases from the early 1990 s, they have delivered major improvements in outcomes. However, despite their demonstrated efficacy, access to biologics worldwide has been limited, often due to their high acquisition cost.^[2]^

As patents for bio-originators expire, they pave the way for the development of biosimilars. These agents demonstrate such a high degree of comparability to bio-originators with respect to quality, safety and efficacy, that they are clinically indistinguishable.^[3]^ Their lower development cost and introduction of competition to the market drives down the cost of biologics, provides billions of dollars of savings in healthcare and, so we are told, further improves patient outcomes by increasing the access to biologics worldwide.^[4]^ To date, 106 biosimilars are approved for use in Europe by the European Medicines Agency (EMA), a number set to steadily rise.^[5]^

Are biosimilars fully delivering on their potential in both the developing and developed world? We suggest not and consider several factors underlie this. For example, whilst a true biosimilar has to pass the most rigorous scrutiny from major regulatory bodies, the so-called “intended copies” or “biomimic” drugs do not, and therefore cannot gain market authorization in developed countries. This prompts the question “How similar does similar really need to be?” It can be argued that if clinical trials report appropriate efficacy and safety of a biomimic, isn’*t* that sufficient? The developed world regulators would say categorically “no” – but less developed economies are starting to rely on such drugs, which are affordable and typically manufactured locally.

Secondly, patients’ and clinicians’ perceptions about bio-similars may play a negative role. Patients sometimes report concerns about the efficacy and side effects of biosimilars, leading to a potential nocebo effect. Similarly, although studies have shown that switching between biosimilars is safe, clinically effective and with no observed increased risk of immunogenicity, the issues of interchangeability, switching, extrapolation and pharmacovigilance remain potential barriers to the use of biosimilars among some healthcare professionals.^[6,7]^

Thirdly, and less well discussed in the literature, whilst the reduced cost of biosimilars should facilitate access to medicines that might otherwise be unaffordable, life is not so straightforward. A challenging example of this can be found in the multisystem autoinflammatory Behçet’s Syndrome (BS), where an effective biologic, interferon alfa-2a, recombinant (Roferon), used with much success worldwide in an off-license capacity, and with the potential advantage of achieving a durable remission, was recently withdrawn for commercial reasons. With no biosimilars available, there remains a frustrating unmet therapeutic need.

Whilst BS is rare in the West, it is prevalent in less developed countries along the Silk Route which runs from Japan, through China and the Middle East, to the Southern Mediterranean. Many of these countries have challenged economies. The two most widely used biologics for BS-infliximab (with many biosimilars) and interferon-alfa 2a (no biosimilars) were studied in a high quality randomized head-to-head controlled trial, which was recently published. The authors reported that both biologics were effective in refractory BS. Unfortunately, just prior to the conclusion of the trial, the manufacturer of the biooriginator interferon-alfa 2a, Roche, discontinued production of this drug.^[8]^

When a bio-originator is discontinued, its biosimilars may still be developed and manufactured, with a fast development process, and substantial savings in acquisition costs. Surely the loss of manufacture of the interferon-alfa 2a biooriginator need not be a problem today, with the potential for biosimilars to meet the therapeutic demand? Sadly, this is not the case. Similarly, a pegylated version of interferon-alfa 2a, with demonstrated benefit in BS, is also not available in the West for BS.^[9]^ Interferon-alfa 2a has been used successfully in various diseases for which it was licensed.^[10]^ It was also widely used successfully off-license, such as in BS, as it was cheaper and, given the prevalence of chronic infections such as viral hepatitis or mycobacterial diseases in Silk Route countries, where infliximab or other tumor necrosis factor (TNF) inhibitors might be relatively contraindicated, it was a most attractive choice. Data on Alpheon was submitted to the EMA in 2003 for consideration as a biosimilar product of interferon-alfa 2a. However, it was not approved for marketing, as both qualitative and quantitative impurity profiles were found during comparability assessment with bio-orginator interferon-alfa 2a. There were also significant differences in virological data, rates between adverse and laboratory-related events and insufficient immunogenicity documentation.^[11]^ It was therefore, not a biosimilar as defined by the EMA or US Food and Drugs Administration (FDA). Since then, the development of various interferon-alfa 2a biosimilars has been undertaken by many companies, including Biosidus, Zydus Cadila, Nanogen, Amega Biotech, PROBIOMED and 3sbio. However, few (if any) have succeeded in producing a biosimilar to EMA or FDA standards- and 3sbio is focusing on the South-East Asian market, without the requirement to gain FDA or EMA approval, and therefore limiting the regions where it can be marketed.

Despite the discontinuation of the bio-originator, the interferon-alfa 2a biosimilar market has the potential for significant growth and demand, due to an increasing prevalence of diseases for which the bio-originator was licensed, such as hepatitis, malignancy and multiple sclerosis. However, for conditions such as BS, where interferon therapy is clearly effective but where the bio-originator did not have a license, a large clinical unmet need remains. At present, a biosimilar cannot be marketed in the West for conditions where the bio-originator is not licensed, the major regulatory bodies have a high bar for approving a new biosimilar and pharmaceutical companies are prone to be risk-averse in developing a drug for particular use off-license. Frustratingly, there does not appear to be a clear path for the introduction of, and hence access to an interferon-alfa 2a biosimilar in the many countries where this is an important drug. This is but one example of the challenge in attempting to meet an unmet therapeutic need, where an effective drug is tantalizingly so near, yet so far.

Whilst biosimilars have transformed the landscape of medicine worldwide, we therefore suggest that they still fall short of meeting their full potential, even within the West. As illustrated by BS, where no biosimilars to interferon-alfa 2a are generally available, a large therapeutic unmet need remains. The issues preventing the successful use of biosimilars, especially around the regulatory process, when there is a lack of bio-originator license must be overcome if we are to fully unlock the full potential benefit of these important agents.
